# Activity of Potassium Channel BmK-NSPK Inhibitor Regulated by Basic Amino Acid Residues: Novel Insight into the Diverse Peptide Pharmacology

**DOI:** 10.3390/molecules30030450

**Published:** 2025-01-21

**Authors:** Zheng Zuo, Xuhua Yang, Haozhen Zhang, Chenhu Qin, Zhijian Cao, Yingliang Wu

**Affiliations:** 1 College of Life Sciences, Wuhan University, Wuhan 430072, China; 2014301060068@whu.edu.cn (Z.Z.); yangxh@whu.edu.cn (X.Y.); zhanghaozhen@whu.edu.cn (H.Z.); 2 Department of Biochemistry and Molecular Biology, College of Basic Medicine, Hubei University of Medicine, Shiyan 442000, China; 2016202040025@whu.edu.cn; 3 National “111” Center for Cellular Regulation and Molecular Pharmaceutics, Key Laboratory of Fermentation Engineering (Ministry of Education), Hubei University of Technology, Wuhan 430072, China

**Keywords:** venomous peptide inhibitor, potassium channel, BmK-NSPK peptide, activity regulation, basic amino acid residue, functional diversity

## Abstract

The molecular interactions between venomous peptides and potassium channels have extensively enriched the knowledge of diverse peptide pharmacology, and the in-depth understanding of general features of the various peptide functions remains a formidable challenge. In this work, the role of peptide basic residues in peptide pharmacology was first investigated. Although the venomous BmK-NSPK peptide had the critically conserved functional residues occurring in its similar and potent potassium channel-inhibiting peptides, it was a remarkably weak inhibitor of potassium channels due to fewer basic residues. Additionally, 1 μM BmK-NSPK only inhibited 1.2 ± 1.0%, 1.7 ± 0.70%, 2.3 ± 0.49% and 5.4 ± 0.70% of hKv1.1, hKv1.2, hKv1.3 and hKv1.6 channel currents. The introduction of one or two basic residues in BmK-NSPK-I15K, BmK-NSPK-I18K, BmK-NSPK-I26K and BmK-NSPK-I18K/I26K could not improve BmK-NSPK activity. The modifications of more than three basic residues were found to continuously improve BmK-NSPK activity, and the corresponding BmK-NSPK-7K and BmK-NSPK-8K mutants could effectively inhibit hKv1.3 channel with IC_50_ values of 2.04 ± 0.68 nM and 21.5 ± 1.99 nM, respectively. Also, 1 μM BmK-NSPK-7K and BmK-NSPK-8K mutants could inhibit 84.1 ± 7.0% and 84.3 ± 1.8% of hKv1.1 channel currents. In addition, BmK-NSPK-7K and BmK-NSPK-8K mutants were found to differentially inhibit hKv1.6 and chimeric hKv1.3 channels. These findings first highlight the critical role of basic residues in the activity of potassium channel peptide inhibitors and provide novel insight into the diverse peptide pharmacology.

## 1. Introduction

With about 100 members, the potassium channels serve a variety of physiological and pathological functions [1]. These channels are widely inhibited by peptide toxins from venomous animals, such as scorpions, snakes, spiders, bees, sea anemones and marine cone snails [2]. These molecular interactions have greatly promoted a remarkable understanding of the structure and function of diverse potassium channels, which was subsequently verified by the potassium channel structures obtained by the X-ray crystallography and cryo-electron microscopy, especially for the channel selectivity filter and turret topology [3,4,5,6,7,8,9,10]. Meanwhile, many peptide toxins and/or analogs have been investigated for treating potassium channel-related diseases [11,12,13,14]. All these progresses strongly indicate that peptide toxins are a remarkably valuable resource for molecular tools and prospective drug candidates acting on the potassium channels. However, the in-depth understanding of general features of the peptide–potassium channel interactions remains a formidable challenge, which is potentially restricting the research and development of peptide toxins nowadays.

**Figure 1:**
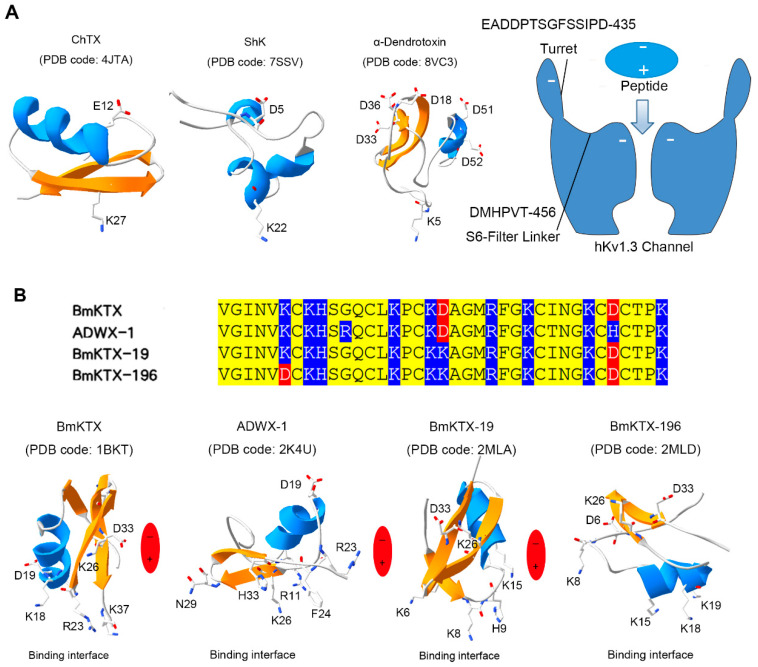
The negatively charged acidic residues orientating peptide binding interfaces via the electrostatic repulsion forces between the negatively charged acidic residues of peptides and potassium channels. (**A**) The representative peptide binding modes and diagram to potassium channels. The binding interfaces of ChTX, ShK and α-Dendrotoxin peptides around the potassium channel pore-blocking basic residues were shown according to the peptide–potassium channel complex structures, respectively [20,21,22]. All the acidic and potassium channel pore-blocking basic residues were labeled in ChTX, ShK and α-Dendrotoxin, respectively. The negative charges were labeled in the outer vestibule of the hKv1.3 model, which is electrostatically associated with the polar peptide model. (**B**) The negatively charged acidic residues orientating the binding interfaces of BmKTX and its three similar analogs. The differential distributions of acidic residues were highlighted by red color in the sequence alignments of BmKTX and its three similar peptides, while the basic and other identical residues were colored by blue and yellow, respectively. The acidic residues in the non-binding interfaces and basic residues in the binding interfaces were labeled in the structures of BmKTX and its three similar peptides according to their structural and functional experiments [27,28,29].

During the molecular interactions between peptides and potassium channels, the most fundamental feature of a pore-blocking basic residue in the peptide toxins has been well confirmed by the site-directed mutagenesis experiments, computation simulation, X-ray crystallography and cryo-electron microscopy. A pore-blocking basic residue in the binding interfaces of various peptide toxins was previously proposed according to extensive experiments from both the peptide and potassium channel mutants. For example, scorpion toxin charybdotoxin (ChTX), sea anemone toxin Shk and snake toxin α-Dendrotoxin might use Lys27, Lys22 and Lys6 to block the pore of potassium channel from the site-directed mutagenesis experiments, respectively [15,16,17]. Later, the docking and molecular dynamic simulations further supported these unique roles of pore-blocking basic residues in scorpion toxin ChTX and sea anemone toxin Shk [15,16,17,18,19]. Nowadays, the peptide toxin–potassium channel complex structures from the X-ray crystallography or cryo-electron microscopy confirmed these pore-blocking basic residues in peptide toxins ChTX, Shk and α-Dendrotoxin [20,21,22] (Figure 1A). As shown by the peptide–potassium channel interaction model in Figure 1A, the polar molecules of these toxins would use their positively charged surface to bind the negatively charged pore region of potassium channels through the classical electrostatic interactions [23,24,25]. Beyond the most fundamental feature of a pore-blocking basic residue in the binding interfaces of different peptide toxins (Figure 1A), some attention has been paid to the diverse binding modes of peptide toxins acting on potassium channels in past years. Theoretically, there are likely more different binding modes of peptide toxins responsible for recognizing the potassium channel since there are a great number of peptide toxins from the different venomous animals. Due to the apparent electrostatic repulsion forces between the negatively charged acidic residues of both peptide toxins and potassium channels in the model of peptide–potassium channel interaction (Figure 1A), the critical role of the negatively charged acidic residue would be helpful to the orientation of peptide binding interfaces, which was first demonstrated by the scorpion toxin BmP05 and its analog BmP05-T with the distinct binding interfaces through regulating the distributions of negatively charged acidic residues [26]. Later, a more classical case was shown by the scorpion toxin BmKTX and its three analogs with distinct distributions of negatively charged acidic residues (Figure 1B). In spite of the high number of similarities with BmKTX and its three analogs, they used four differential molecular surfaces as their own binding interfaces, which well utilized the electrostatic repulsion forces between the negatively charged acidic residues of both peptide toxins and potassium channels [27,28,29]. This progress would accelerate our understanding of the role of negatively charged acidic residues in the orientation of diverse peptide binding interfaces.

Figuratively, the negatively and positively charged residues of peptide toxins look similar to the two sides of the same coin. Apart from the negatively charged residues, the role of positively charged residues in the orientation of the peptide binding interface was first investigated in this work. Scorpion toxin BmK-NSPK, with the same distributions of negatively charged acidic residues as other similar potent peptide inhibitors of potassium channels [30], was found not to inhibit the human Kv1.3 potassium channel, which is a highly classical target of numerous peptide inhibitors [31,32,33]. In view of the fewer positively charged basic residues, the gradual introduction of more positively charged basic residues was intriguingly found to increase the activities of BmK-NSPK peptide analogs acting on Kv1.3 and other potassium channels. These findings first revealed the novel role of positively charged basic residues in regulating the peptide activity, which further provided insight into the diverse pharmacology of a great number of peptide toxins from various venomous animals.

## 2. Results

### 2.1. Similar Structure and Functional Loss of BmK-NSPK Peptide Due to Fewer Basic Residues

**Figure 2:**
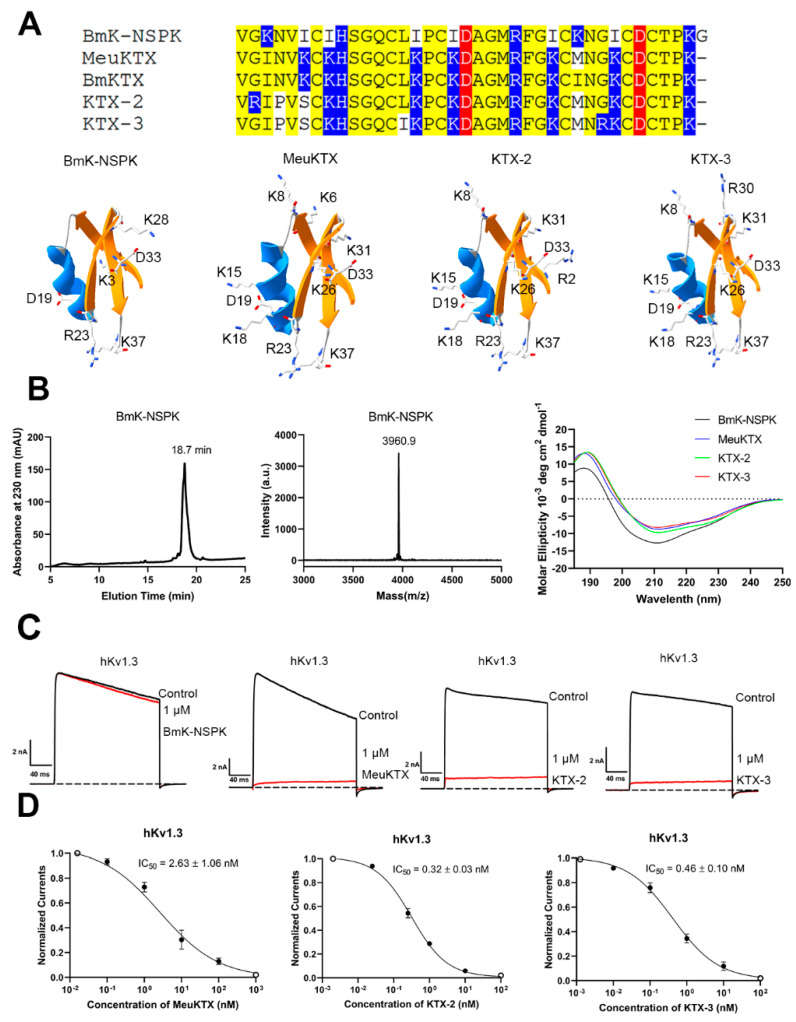
Structural similarities and functional differences between BmK-NSPK and its similar analogous peptides acting on hKv1.3 channels. (**A**) Identical distributions of acidic residues and differential distributions of basic residues were shown in the sequences and structures of BmK-NSPK, MeuKTX, BmKTX, KTX-2 and KTX-3 peptides, the acidic residues, basic residues and other identical residues were colored by red, blue and yellow, respectively. The structures of BmK-NSPK, MeuKTX and KTX-3 are modeled using the BmKTX structure (PDB code: 1BKT) as a template. (**B**) The production and structural analysis of recombinant BmK-NSPK, MeuKTX, KTX-2 and KTX-3 peptides. The chromatographic purification and molecular mass detection of representative BmK-NSPK peptides were shown. The circular dichroism spectra of four BmK-NSPK, MeuKTX, KTX-2 and KTX-3 peptides were measured by the circular dichroism spectrometry. (**C**) Differential inhibitory effects of BmK-NSPK and its similar peptides. The representative current traces of 1 μM BmK-NSPK, MeuKTX, KTX-2 and KTX-3 on hKv1.3 channels were shown. (**D**) Average normalized current inhibition by different concentrations of MeuKTX, KTX-2 and KTX-3 for hKv1.3 channels with IC_50_ values of 2.63 ± 1.06 nM, 0.32 ± 0.03 nM and 0.46 ± 0.10 nM, respectively. Each channel was tested at least three times (n ≥ 3). The results are shown as the mean ± SE.

BmK-NSPK peptide was purified from scorpion venom [30]. In spite of the highly structural similarity between the BmK-NSPK and BmKTX-like peptides, the sequence and structure analysis indicated that it had two acidic Asp19 and Asp33 residues, which also occurred in the BmKTX-like peptides, including MeuKTX, BmKTX, KTX-2 and KTX-3 peptides. However, there were much fewer basic residues in BmK-NSPK than MeuKTX, BmKTX, KTX-2 and KTX-3 (Figure 2A). Since the peptide polarity is mainly defined by the negatively charged and positively charged residues (Figure 1), the molecular polarity of the BmK-NSPK peptide would be different from those in MeuKTX, BmKTX, KTX-2 and KTX-3, which likely affected the pharmacological function of BmK-NSPK peptide.

In order to verify the differential pharmacological activities between BmK-NSPK, MeuKTX, KTX-2 and KTX-3, their recombinant peptides were produced according to our previous procedure [34,35]. As shown in Figure 2B, the representative BmK-NSPK peptide was obtained through high-performance liquid chromatography by manual collection at 18–20 min. Then, MALDI-TOF-MS was further used to analyze BmK-NSPK. The determined molecular mass was 3960.9 Da, which well corresponds to its calculated value of 3659.8 Da. The circular dichroism (CD) spectra also indicated the overall similarity to the secondary structures between BmK-NSPK, MeuKTX, KTX-2 and KTX-3 peptides. These results showed that BmK-NSPK, MeuKTX, KTX-2 and KTX-3 peptides were successfully produced. Subsequently, the patch clamp technique was used to characterize the pharmacological activities of BmK-NSPK, MeuKTX, KTX-2 and KTX-3 peptides according to our previously described procedure [36,37,38]. Since the Kv1.3 channel shows an unusually broad sensitivity to peptide toxins from venomous animals [2], the human Kv1.3 (hKv1.3) channel was expressed in HEK293T cells, and the effects of 1 μM peptides on hKv1.3 channel currents were measured. Pharmacological data indicated that 1 μM BmK-NSPK, MeuKTX, KTX-2 and KTX-3 peptides were able to inhibit 2.3 ± 0.49%, 92.6 ± 2.9%, 94.9 ± 2.6% and 91.6 ± 3.3% of potassium currents mediated by hKv1.3 channel, respectively (Figure 2C). The concentration-dependent experiments showed that MeuKTX, KTX-2 and KTX-3 peptides inhibited hKv1.3 channel currents with IC_50_ values of 2.63 ± 1.06 nM, 0.32 ± 0.03 nM and 0.46 ± 0.10 nM, respectively (Figure 2C). The functional loss of BmK-NSPK revealed that the fewer number of basic amino acids significantly affected its pharmacology function in the presence of high sequence and structure similarity between BmK-NSPK, MeuKTX, KTX-2 and KTX-3 peptides.

### 2.2. Less Effect of One and Two Basic Residue Introduction on BmK-NSPK Function

**Figure 3:**
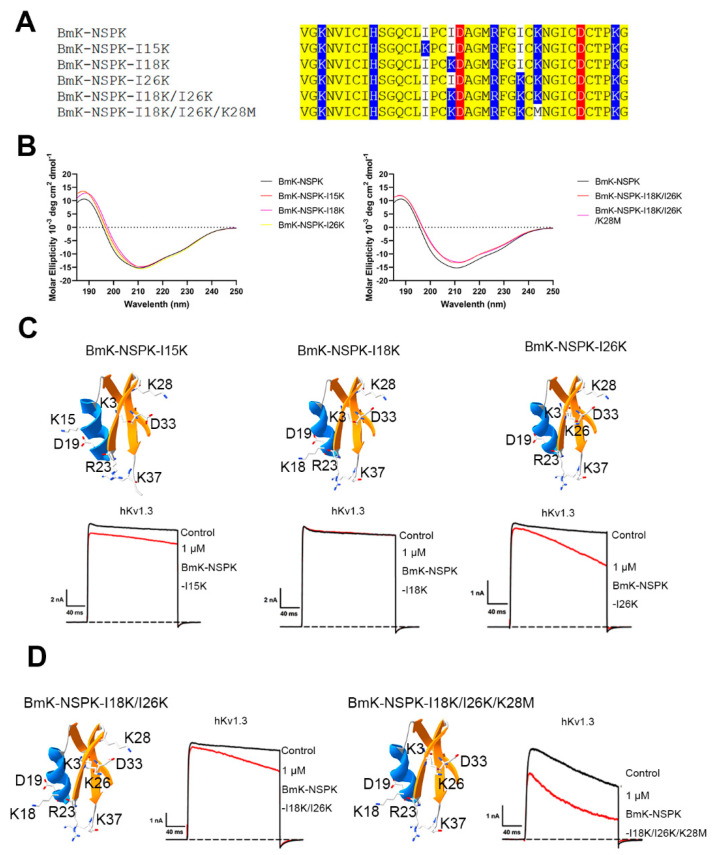
The introduction of one and two basic residues showing the minor effects on BmK-NSPK activity. (**A**) Sequence alignments of BmK-NSPK and its mutants with one and two more basic residues, the acidic residues, basic residues and other identical residues were colored by red, blue and yellow, respectively. (**B**) Circular dichroism spectra of BmK-NSPK and its different mutants. (**C**) The structures and inhibitory effects of BmK-NSPK mutants with one more basic residue. The distributions of all basic residues were shown in the BmK-NSPK mutant structures. The representative current traces of 1 μM BmK-NSPK mutants on hKv1.3 channels were colored by red lines. (**D**) The structures and inhibitory effects of BmK-NSPK-I18K/I26K and BmK-NSPK-I18K/I26K/K28M mutants. The distributions of all basic residues were shown in the BmK-NSPK mutant structures. The representative current traces of 1 μM BmK-NSPK-I18K/I26K and BmK-NSPK-I18K/I26K/K28M mutants on hKv1.3 channels were colored by red lines. Each channel was tested at least three times (n ≥ 3). The results are shown as the mean ± SE.

In order to regulate BmK-NSPK activity towards the hKv1.3 channel, the strategy of basic residue introduction was adopted in this work. Firstly, the effect of a single basic residue introduction was investigated on the BmK-NSPK function. As shown in Figure 3A, three BmK-NSPK-I15K, BmK-NSPK-I18K and BmK-NSPK-I26K mutants were constructed and produced since these lysine residues occurred in BmK-NSPK-like MeuKTX, KTX-2 and KTX-3 peptides. Although the introduction of a single basic residue did not affect the overall conformation of BmK-NSPK peptide (Figure 3B), these three mutants did not effectively inhibit hKv1.3 channel currents even at 1 μM concentration (Figure 3C). Subsequently, the introduction of two basic residues by constructing a BmK-NSPK-I18K/I26K mutant did not change the overall conformation of the BmK-NSPK peptide (Figure 3B) and could not improve BmK-NSPK activity (Figure 3D). In view of the fact that Lys28 residue in BmK-NSPK did not appear in MeuKTX, KTX-2 and KTX-3 peptides (Figure 2A), a new BmK-NSPK-I18K/I26K/K28M mutant was further produced and found to maintain the overall conformation of BmK-NSPK peptide (Figure 3A,B). Interestingly, 1 μM BmK-NSPK-I18K/I26K/K28M was able to inhibit 26.5 ± 0.60% of hKv1.3 channel currents (Figure 3D). In comparison with the BmK-NSPK-I18K/I26K mutant, the improvement of BmK-NSPK-I18K/I26K/K28M function illustrated that the modification of Lys28 in BmK-NSPK could change the peptide polarity and affect the peptide binding to hKv1.3 channel. These data preliminarily illustrated a certain role of basic amino acid residues in BmK-NSPK pharmacology.

### 2.3. Significant Effect of Several Basic Residue Introduction on BmK-NSPK Function

**Figure 4:**
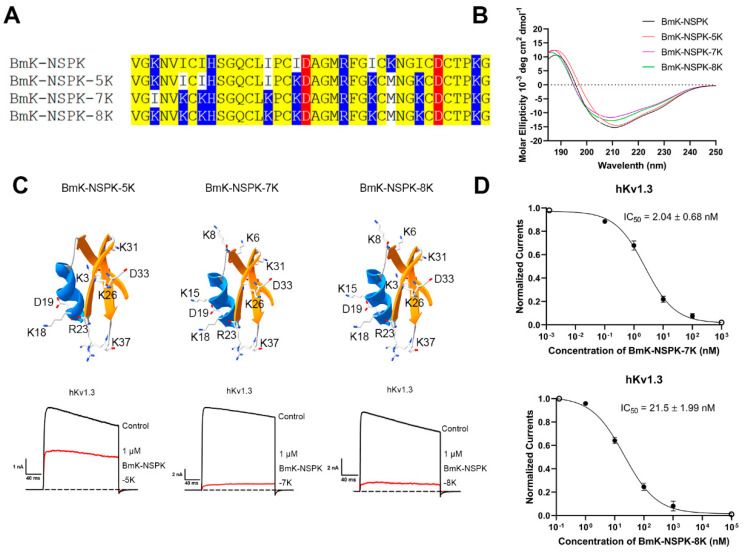
The introduction of several basic residues indicating the remarkable effects on BmK-NSPK activity. (**A**) Sequence alignments of BmK-NSPK and its mutants with the introduction of several basic residues, the acidic residues, basic residues and other identical residues were colored by red, blue and yellow, respectively. (**B**) Circular dichroism spectra of BmK-NSPK, BmK-NSPK-5K, BmK-NSPK-7K and BmK-NSPK-8K mutants. (**C**) The structures and inhibitory effects of BmK-NSPK mutants with the introduction of several basic residues. The distributions of the introduction of all basic residues were shown in the BmK-NSPK mutant structures. The representative current traces of 1 μM BmK-NSPK mutants on hKv1.3 channels were colored by red lines. (**D**) Average normalized current inhibition by different concentrations of BmK-NSPK-7K and BmK-NSPK-8K for the hKv1.3 channel with IC_50_ values of 2.04 ± 0.68 nM and 21.5 ± 1.99 nM, respectively. Each channel was tested at least three times (n ≥ 3). The results are shown as the mean ± SE.

Overall, BmK-NSPK had several basic residues fewer than MeuKTX, KTX-2 and KTX-3 peptides (Figure 2A) so that one or two basic residue introductions were possibly difficult to improve BmK-NSPK activity (Figure 3). In order to confirm this inference, a new mutant BmK-NSPK-5K, named by having a total of five lysine residues in the peptide, was produced and also maintained the overall conformation of BmK-NSPK peptide (Figure 4A,B). In comparison with the 26.5 ± 0.60% inhibition of hKv1.3 channel currents by 1 μM of the BmK-NSPK-I18K/I26K/K28M mutant, the additional introduction of Lys31 in BmK-NSPK-5K further increased the peptide activity, which was shown by the 50.6 ± 0.84% inhibition of hKv1.3 channel currents by 1 μM BmK-NSPK-5K (Figure 4C). Based on the functional improvement of BmK-NSPK-5K peptide, BmK-NSPK-7K and BmK-NSPK-8K mutants were designed while their conformations were found to be similar to that of BmK-NSPK peptide (Figure 4A,B). Pharmacological experiments indicated that 1 μM BmK-NSPK-7K and BmK-NSPK-8K could remarkably inhibit 90.2 ± 1.5% and 89.6 ± 2.1% of hKv1.3 channel currents, respectively (Figure 4C). The concentration-dependent experiments showed that BmK-NSPK-7K and BmK-NSPK-8K peptides inhibited hKv1.3 channel currents with IC_50_ values of 2.04 ± 0.68 nM and 21.5 ± 1.99 nM, respectively (Figure 4D). Interestingly, BmK-NSPK-8K showed ~10-fold decreased activity compared with BmK-NSPK-7K after the addition of a single Lys3 residue, which further highlighted the important role of basic amino acid residue in regulating peptide pharmacology. These results definitely illustrated the critical effect of basic amino acid residues on BmK-NSPK pharmacology.

### 2.4. Importance of Basic Residue on BmK-NSPK Inhibition of Other Potassium Channels

**Figure 5:**
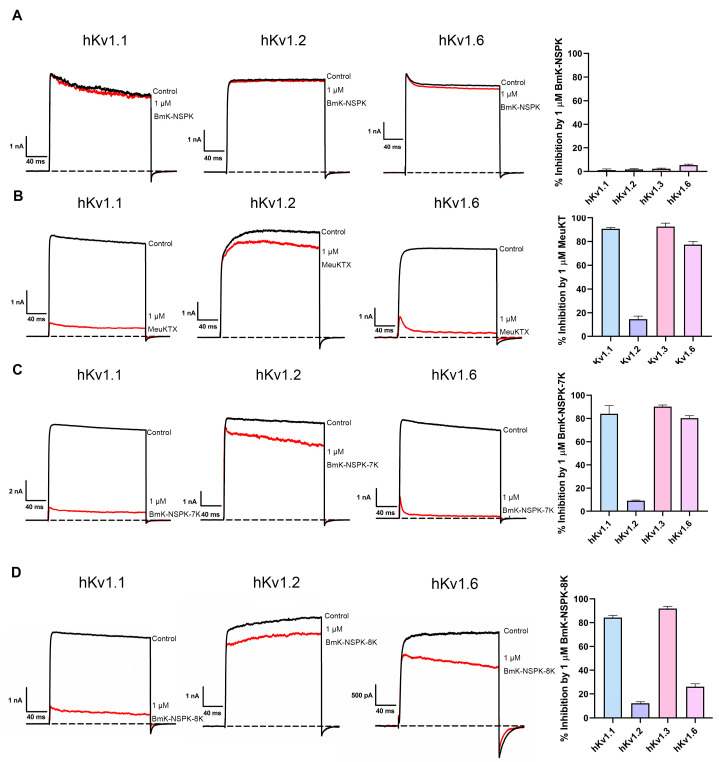
Differential inhibitory effects of 1 μM BmK-NSPK, MeuKTX, BmK-NSPK-7K and BmK-NSPK-8K on the different potassium channels. (**A**–**D**) Representative current traces of 1 μM BmK-NSPK, MeuKTX, BmK-NSPK-7K and BmK-NSPK-8K on hKv1.1, hKv1.2 and hKv1.6 channels. (**A**) Then, 1 μM BmK-NSPK inhibited potassium currents of 1.2 ± 1.0% for hKv1.1 channel, 1.7 ± 0.70% for hKv1.2 channel, 2.3 ± 0.49% for hKv1.3 channel and 5.4 ± 0.70% for hKv1.6 channel, respectively. (**B**) Also, 1 μM MeuKTX inhibited potassium currents of 90.7 ± 1.1% for hKv1.1 channel, 14.6 ± 2.6% for hKv1.2 channel, 92.6 ± 2.9% for hKv1.3 channel and 77.4 ± 2.7% for hKv1.6 channel, respectively. (**C**) Next, 1 μM BmK-NSPK-7K inhibited potassium currents of 84.1 ± 7.0% for hKv1.1 channel, 9.2 ± 0.6% for hKv1.2 channel, 90.2 ± 1.5% for hKv1.3 channel and 79.2 ± 2.4% for hKv1.6 channel, respectively. (**D**) Finally, 1 μM BmK-NSPK-8K inhibited potassium currents of 84.3 ± 1.8% for hKv1.1 channel, 12.3 ± 1.8% for hKv1.2 channel, 89.6 ± 2.1% for hKv1.3 channel and 34.1 ± 4.8% for hKv1.6 channel, respectively. Each channel was tested at least three times (n ≥ 3). The results are shown as the mean ± SE.

Besides the activity improvement of BmK-NSPK analogs acting on the hKv1.3 channel after increasing the number of basic residues, the effect was also investigated on the potency of BmK-NSPK analogs acting on other potassium channels. Similarly to the minor effect of 1 μM BmK-NSPK on hKv1.3 channel currents (Figure 2C), less inhibition of hKv1.1, hKv1.2 and hKv1.6 channel currents also occurred in the presence of 1 μM BmK-NSPK (Figure 5A).

As for potent Kv1.3 channel-inhibiting BmK-NSPK-like MeuKTX (Figure 2C), the hKv1.2 channel was also found to be weakly inhibited by 1 μM MeuKTX (Figure 5B). However, both hKv1.1 and hKv1.6 channels were significantly inhibited by MeuKTX. As shown in Figure 5B, 90.7 ± 1.1% of hKv1.1 channel currents and 77.4 ± 2.7% of hKv1.6 channel currents could be inhibited by 1 μM MeuKTX, respectively. These pharmacological differences between BmK-NSPK and MeuKTX peptides again indicated that the fewer basic residues also played an important role in regulating peptide potencies on hKv1.1 and hKv1.6 channels. Through increasing the number of basic residues in BmK-NSPK, 1 μM BmK-NSPK-7K and BmK-NSPK-8K was also found to inhibit 84.1 ± 7.0% and 84.3 ± 1.8% of hKv1.1 channel currents and 79.2 ± 2.4% and 34.1 ± 4.8% of hKv1.6 channel currents, while they showed weak effects on hKv1.2 channel, which highlighted the essential role of basic residue number on the BmK-NSPK pharmacology (Figure 5C,D). In addition, the differential potencies between BmK-NSPK-7K and BmK-NSPK-8K acting on the hKv1.6 channel further illustrate the importance of basic residue number in peptide pharmacology. Together, the introduction of more basic residues could render less active BmK-NSPK peptides as potent inhibitors of different potassium channels.

### 2.5. Differential Effects of Basic Residue Introduction on BmK-NSPK Inhibition of Potassium Channel Chimeras

**Figure 6:**
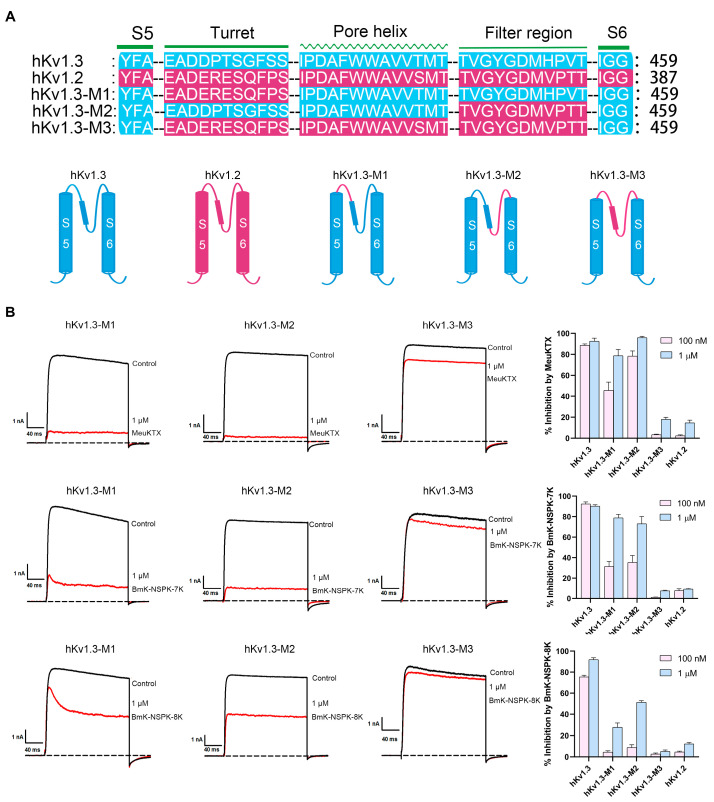
Inhibitory effects of MeuKTX, BmK-NSPK-7K and BmK-NSPK-8K on hKv1.3 channel chimeras. (**A**) Sequence alignments for pore regions of the hKv1.3, hKv1.2 and mutant channels. Abridged general views of the pore region of hKv1.3, hKv1.2 and hKv1.3 channel chimeras. (**B**) Representative current traces of 1 μM MeuKTX, BmK-NSPK-7K and BmK-NSPK-8K on hKv1.3 channel chimeras. The 100 nM MeuKTX inhibited hKv1.3 channel chimera currents of 45.7 ± 7.9% for hKv1.3-M1 channel, 78.3 ± 4.9% for hKv1.3-M2 channel, 3.9 ± 0.21% for hKv1.3-M3 channel, respectively. The 1 μM MeuKTX inhibited hKv1.3 channel chimera currents of 82.8 ± 6.1% for hKv1.3-M1 channel, 95.9 ± 1.3% for hKv1.3-M2 channel, 16.1 ± 2.0% for hKv1.3-M3 channel, respectively. Also, 100 nM BmK-NSPK-7K inhibited hKv1.3 channel chimera currents of 23.4 ± 3.4% for hKv1.3-M1 channel, 35.4 ± 6.7% for hKv1.3-M2 channel, 1.2 ± 0.21% for hKv1.3-M3 channel, respectively. Next, 1 μM BmK-NSPK-7K inhibited hKv1.3 channel chimera currents of 78.8 ± 3.4% for hKv1.3-M1 channel, 72.9 ± 7.0% for hKv1.3-M2 channel, 7.4 ± 0.64% for hKv1.3-M3 channel, respectively. Additionally, 100 nM BmK-NSPK-8K inhibited hKv1.3 channel chimera currents of 4.5 ± 1.2% for hKv1.3-M1 channel, 8.8 ± 2.6% for hKv1.3-M2 channel, 2.7 ± 0.92% for hKv1.3-M3 channel, respectively. Finally, 1 μM BmK-NSPK-8K inhibited hKv1.3 channel chimera currents of 27.7 ± 4.0% for hKv1.3-M1 channel, 51.3 ± 1.5% for hKv1.3-M2 channel, 5.1± 1.4% for hKv1.3-M3 channel, respectively. Each channel was tested at least three times (n ≥ 3). The results are shown as the mean ± SE.

In order to further investigate the effect of basic residue number on BmK-NSPK pharmacology, the activities of BmK-NSPK analogs were also tested on the Kv1.3 channel chimeras in this work. As shown in Figure 1A, the two domains responsible for peptide binding are the turret and filter region in the potassium channels. Based on the weak inhibition potencies of BmK-NSPK-7K and BmK-NSPK-8K towards hKv1.2 channel (Figure 5C,D), the work first constructed the chimeric hKv1.3-M1, in which the turret domain of hKv1.3 channel was substituted by the equivalent region of hKv1.2 channel (Figure 6A). Pharmacological experiments with 100 nM and 1 μM MeuKTX were able to inhibit 45.7 ± 7.9% and 82.8 ± 6.1% of hKv1.3-M1 channel currents, respectively (Figure 6B). Compared with the corresponding 88.6 ± 1.4% and 92.6 ± 2.9% of hKv1.3 channel currents, these weaker inhibition potencies on hKv1.3-M1 channels indicated the important role of channel turret domain in affecting MeuKTX binding (Figure 6B). The differential inhibitory activities further occurred in BmK-NSPK analogs, and 100 nM and 1 μM BmK-NSPK-7K, respectively, showed the 23.4 ± 3.4% and 78.8 ± 3.4% inhibition of hKv1.3-M1 channel currents, which was remarkably stronger than corresponding 4.5 ± 1.2% and 27.7 ± 4.0% inhibition of hKv1.3-M1 channel currents by 100 nM and 1 μM BmK-NSPK-8K, respectively (Figure 6B). These data indicated the important effects of basic residue number on BmK-NSPK pharmacology on the chimeric hKv1.3-M1 channel.

Apart from the chimeric hKv1.3-M1, this work further constructed the chimeric hKv1.3-M2 channel, in which the filter region of hKv1.3 channel was substituted by the equivalent region of hKv1.2 channel (Figure 6A). Pharmacological experiments 100 nM and 1 μM MeuKTX were able to inhibit 78.3 ± 4.9% and 95.9 ± 1.3% of hKv1.3-M2 channel currents, respectively, which indicated the minor effect of channel filter region on MeuKTX binding (Figure 6B). However, more significant effects were also observed for BmK-NSPK analogs with different numbers of basic residues. Also, 100 nM and 1 μM BmK-NSPK-7K, respectively, blocked 35.4 ± 6.7% and 72.9 ± 7.0% currents of hKv1.3-M2 channel, which was more significantly potent than corresponding 8.8 ± 2.6% and 51.3 ± 1.5% inhibition of hKv1.3-M2 channel currents by 100 nM and 1 μM BmK-NSPK-8K, respectively (Figure 6B). These data again illustrated the important effects of basic residue number on BmK-NSPK pharmacology on the chimeric hKv1.3-M2 channel.

When all the turrets, pore helixes and filter regions of hKv1.3 channel were replaced by the corresponding regions of hKv1.2 channel, it was understandable that the resulting chimeric hKv1.3-M3 channel was weakly inhibited by MeuKTX, BmK-NSPK-7K and BmK-NSPK-8K peptides, which resembled the inhibitory performances of hKv1.2 channel by these peptides (Figure 6).

## 3. Discussion

In previous decades, a great number of potassium channel inhibitors have been discovered through multiple techniques from different venomous animals [2,39]. Beyond the well-known feature of a channel pore-blocking basic residue in the binding interfaces of various peptide toxins [6,15,16,17,18,19,20,21,22], the in-depth insight into the general feature of the peptide–potassium channel interactions remains a formidable challenge.

Based on the theory of polar molecular interactions (Figure 1A), the molecular polarity of peptide toxins, usually determined by the widely distributed positively charged basic residues and characteristically distributed negatively charged acidic residues, was found to be able to regulated by changing the distributions of a few acidic residues, which was illustrated by orientating the various binding interfaces of BmP05, BmKTX and their analogous toxins [26,27,28,29] (Figure 1B). Figuratively, the negatively and positively charged residues of peptide toxins look similar to the two sides of the same coin. In this work, the role of basic residues in peptide toxin pharmacology was first investigated by the example of natural scorpion toxin BmK-NSPK [30]. As shown in Figure 2A, the BmK-NSPK sequence is remarkably similar to those of MeuKTX, BmKTX, KTX2 and KTX3 except for fewer basic residues. Compared to the functional Lys18, Arg23, Phe24, Lys26 and Lys37 residues in BmKTX peptide from the previous mutagenesis and computational simulation experiments [27,29], BmK-NSPK peptides had the more important Arg23, Phe24 and Lys37 residues, and only lost less important Lys18 and Lys26 residues. Pharmacological experiments showed that 1 μM BmK-NSPK only inhibited 2.3 ± 0.49% of hKv1.3 channel currents while 1 μM MeuKTX, KTX-2 and KTX-3, with the same functional residues of BmKTX peptide, significantly inhibited 92.6 ± 2.9%, 94.9 ± 2.6%, 91.6 ± 3.3% of hKv1.3 channel currents, respectively (Figure 2). Such remarkably weak pharmacological activity of the BmK-NSPK peptide was clearly related to its fewer basic residues according to the theory of polar molecular interactions (Figure 1). Based on the classical analysis of protein structure–function relationship [40,41,42,43], the introduction of single Lys18 or Lys26 or both of them could endow BmK-NSPK with the same functional residues as those of BmKTX, MeuKTX, KTX-2 and KTX-3 (Figure 3A,B). However, these substitutions also could not improve the inhibitory activity of the BmK-NSPK peptide on the hKv1.3 channel (Figure 3C,D), which further indicated that the distribution of basic residue likely affected the BmK-NSPK function. On the basis of the BmK-NSPK-I18K/I26K mutant, an additional modification of the basic residue distribution in BmK-NSPK-I18K/I26K/K28M mutant was found to increase BmK-NSPK activity with the 26.5 ± 0.60% inhibition of hKv1.3 channel currents by 1 μM concentration (Figure 3A,D), which showed the important role of the basic residue distribution in BmK-NSPK pharmacology. In order to illustrate the critical role of the basic residue distribution in the BmK-NSPK function, the Lys31 was further introduced in the BmK-NSPK-I18K/I26K/K28M mutant, and the resulting BmK-NSPK-5K mutant was more potent than the BmK-NSPK-I18K/I26K/K28M mutant (Figure 4A,C). Subsequently, the more introductions of Lys6, Lys8 and Lys15 without Lys3 or with Lys3 residues in BmK-NSPK-5K made BmK-NSPK become the potent inhibitors of hKv1.3 channel with IC_50_ values of 2.04 ± 0.68 nM and 21.5 ± 1.99 nM for BmK-NSPK-7K and BmK-NSPK-8K, respectively (Figure 4C,D). The functional changes in various BmK-NSPK mutants acting in the hKv1.3 channel highlighted the critical role of the number and distribution modification of basic residues in BmK-NSPK pharmacology. Structurally, these different numbers of the basic residues among Lys3, Lys6, Lys8, Lys15, Lys28 and Lys31, differentially distributed above the line connecting Asp19 and Asp33 while the same Lys18, Arg23, Lys26 and Lys37 basic residues appeared under the line connecting Asp19 and Asp33 among BmK-NSPK-I18K/I26K, BmK-NSPK-I18K/I26K/K28M, BmK-NSPK-5K, BmK-NSPK-7K and BmK-NSPK-8K, which resulted into their differential molecular polarities and pharmacological activities (Figure 3D and Figure 4C). These findings indicated that the functional roles of sequence-conserved residues depended on the molecular polarity in various potassium channel-inhibiting peptides since the peptide molecular polarity was affected by the distribution feature of basic residues.

Apart from the pharmacological characterization of BmK-NSPK analogs acting on the hKv1.3 channel, the effects of BmK-NSPK-7K and BmK-NSPK-8K mutants on other potassium channels and chimeric hKv1.3 channels were also investigated. Among the representative hKv1.1, hKv1.2 and hKv1.6 channels, 1 μM BmK-NSPK-7K and BmK-NSPK-8K mutants, respectively, inhibited 79.2 ± 2.4% and 34.1 ± 4.8% of hKv1.6 channel currents (Figure 5C,D), which showed the important role of the basic residue number in the peptide functions when they bound a specific potassium channel. Interestingly, such phenomena also obviously appeared for BmK-NSPK-7K and BmK-NSPK-8K mutants acting on chimeric hKv1.3 channels. Both 100 nM and 1 μM BmK-NSPK-7K showed stronger inhibition of chimeric hKv1.3-M1 and hKv1.3-M2 channel currents than those of 100 nM and 1 μM BmK-NSPK-8K (Figure 6). These results further indicated the important role of basic residue number and distribution in peptide pharmacology.

## 4. Method and Materials

### 4.1. Construction of Expression Vectors for Fusion Proteins

The peptide sequences of MeuKTX, KTX-2, KTX-3 and BmK-NSPK were all obtained from NCBI. Using the classical overlapping PCR strategy, the DNA fragments encoding MeuKTX, KTX-2, KTX-3 and BmK-NSPK were obtained, respectively. These DNA fragments were then inserted into the expression vector pET-32a. Recombinant expression vectors of BmK-NSPK mutants were generated via directed mutagenesis based on the BmK-NSPK expression vector. Before the plasmid expression, the recombinant plasmids were verified through DNA sequencing.

### 4.2. Expression, Purification and Identification of Peptides

Firstly, the recombinant expression vectors encoding proteins were introduced into *E. coli* Rosetta (DE3) cells. The transformed bacterial cultures were maintained in an LB medium supplemented with 50 μg/mL ampicillin at 37 °C until the OD_630_ reading was between 0.4 and 0.6. Then, the cultures were induced for protein expression by the addition of 1 mM isopropyl β-D-thiogalactopyranoside (IPTG) and further incubated at 25 °C for 12 to 15 h. The bacterial cells were harvested, resuspended in 20 mM imidazole buffer (pH 8.0) at 4 °C and lysed through the ultrasonicator. The resulting fusion proteins were purified using Ni^2+^ affinity chromatography, after which the His tag was cleaved using recombinant enterokinase (Beyotime Biotech, Shanghai, China) at 25 °C for 12 h. The digested proteins were then further purified and isolated using high-performance liquid chromatography (HPLC) on a C18 column (10 × 250 mm, 5 µm), employing a linear gradient of 5 to 95% acetonitrile with 0.1% trifluoroacetic acid (TFA) over 60 min at a constant flow rate of 4 mL/min. The purified peptides were identified using matrix-assisted laser desorption/ionization time-of-flight mass spectrometry (MALDI-TOF-MS).

### 4.3. Circular Dichroism (CD) Spectroscopy

All secondary structures of purified peptides were analyzed by CD spectroscopy. The samples were dissolved in water at approximately 0.1 mg/mL. The spectrum data were gathered at 25 °C, spanning over wavelengths from 180 to 260 nm on a Chirascan™V100 (Applied Photophysics, Leatherhead, Surrey, UK). The CD spectra were averaged from three repetitions, with the blank spectrum for water subtracted. The results were displayed as mean residue weight (MRW) molar ellipticity [θ] (deg·cm^2^·dmol^−1^).

### 4.4. Structural Analysis of Peptides

The 3D structures of MeuKTX, KTX-3 and BmK-NSPK, and the mutants of BmK-NSPK were all modeled by the SWISS-MODEL server (https://swissmodel.expasy.org/, accessed on 11 August 2024). The structural diagram of peptides was drawn by Swiss-PdbViewer software (version 4.1.0).

### 4.5. Potassium Channel Expression Vectors

The pRc/CMV-hKv1.3 was kindly provided by Professor Stephan Grissmer (University of Ulm, Ulm, Germany) and Professor Olaf Pongs (Zentrum für Molekulare Neurobiologie der Universität Hamburg, Hamburg, Germany). The cDNAs encoding hKv1.1 and hKv1.2 (from Professor Stephan Grissmer, University of Ulm, Ulm, Germany) were subcloned into the vector pIRES2-EGFP (TaKaRa Clontech, Mountain View, CA, USA). The cDNAs encoding hKv1.6 were subcloned into the vector pcDNA 3.1(+) (TaKaRa Clontech, Mountain View, CA, USA). A QuikChange Site-Directed Mutagenesis Kit (Stratagene, La Jolla, CA, USA) was used to produce the mutants based on the wild-type hKv1.3 channel plasmids. All plasmids and their mutants were verified by DNA sequencing before the expression. The three channel chimeras were, respectively, named by hKv1.3-M1 (425DPTSGFS432 → 425ERESQFP432), hKv1.3-M2 (452HPV456 → 452VPT456) and hKv1.3-M3 (425DPTSGFS432 → 425ERESQFP432, 452HPV456 → 452VPT456).

### 4.6. Cell Culture and Transfection

In a 5% CO_2_ incubator at 37 °C, HEK293T cells were cultured in DMEM medium (Gibco, Grand Island, NY, USA) supplemented with 10% fetal bovine serum and 1% penicillin/streptomycin. Potassium channel plasmids and pEGFP-N1 were co-transfected into the HEK293 cells using the ExFect Transfection Reagent (Vazyme, Nanjin, China). After one day of transfection, the potassium currents were recorded, and the positive cells were selected based on the presence of GFP fluorescence.

### 4.7. Electrophysiological Recordings

Whole-cell current measurement and data acquisition were performed using an EPC-10 patch-clamp amplifier controlled by Pulse (HEKA Elektronik, Lambrecht, Germany). The potassium currents were recorded according to the recording protocol for the Kv1 channel, as described in previously published references [35,36]. Peptides were dissolved in an external solution containing 0.01% BSA for electrophysiological experiments. The patch pipette solution contained 140 mM KF (Sigma, St. Louis, MC, USA), 2 mM MgCl_2_ (Sinopharm Chemical Reagent Co., Ltd., Shanghai, China), 1 mM EGTA (Sigma) and 10 mM HEPES (neoFroxx, Einhausen, Hessen, Germany) (pH adjusted to 7.4 with KOH (Sinopharm Chemical Reagent Co., Ltd., Shanghai, China)), and the bath solution contained 4.5 mM KCl, 160 Mm NaCl (Sinopharm Chemical Reagent Co., Ltd., Shanghai, China), 5 mM HEPES, 2 mM CaCl_2_ (Sinopharm Chemical Reagent Co., Ltd., Shanghai, China) and 1 mM MgCl_2_ (pH adjusted to 7.4 with NaOH (Sinopharm Chemical Reagent Co., Ltd., Shanghai, China)).

### 4.8. Data Analysis

Graphing and data analysis were conducted using GraphPad Prism software (Version 8.0, San Diego, CA, USA), ClampFit software (version 10.2, Molecular Devices, Sunnyvale, CA, USA), and SigmaPlot software (version 12.0, IBM SPSS, Chicago, IL, USA). The dose–response relationship between the channel current and peptide inhibition was modeled by the modified Hill equation: I_peptide_/I_control_ = 1/{1 + [peptide]/IC_50_}, where I_peptide_ represents the potassium current under peptide inhibition, I_control_ represents the peak current, IC_50_ is the half-maximal inhibition concentration and [peptide] represents the peptide concentration. The IC_50_ was obtained by fitting the Hill equation. Results are presented as mean ± SE, with each sample tested on at least three cells (n ≥ 3).

## 5. Conclusions

The pharmacological activity of the BmK-NSPK inhibitor was found to be regulated by modifying the distribution of basic amino acid residues. Although the BmK-NSPK peptide weakly inhibited the hKv1.1, hKv1.3 and hKv1.6 channels due to fewer basic residues, its activity was able to be gradually increased through the introduction of basic residues. These findings first highlight the critical role of basic residues in the functions of potassium channel peptide inhibitors, provide novel insight into the diverse peptide pharmacology, and further accelerate the research and development of peptide inhibitors as potential drugs in the future.

## Data Availability

Data are contained within the article.
